# Relationship between gut microbiota and nutritional status in patients on peritoneal dialysis

**DOI:** 10.1038/s41598-023-27919-3

**Published:** 2023-01-28

**Authors:** Na Tian, Yinyin Yan, Na Chen, Siyao Xu, Rui Chu, Mengting Wang, Surong Duan, Hongyan Ren, Shuhua Song, Li Wang, Xiaoqin Ma, Mei Xu, Li Na, Menghua Chen, Philip Kam-Tao Li

**Affiliations:** 1 Department of Nephrology, General Hospital of Ningxia Medical University, No.804 Shengli Road, Yinchuan, 750004 Ningxia China; 2 Department of Respiratory Medicine, Hanzhong Central Hospital, Shanxi, 723000 China; 3 Shanghai Mobio Biomedical Technology Co. Ltd., Shanghai, 201100 China; 4 BioBank of General Hospital of Ningxia Medical University, Ningxia, 750004 China; 5 Department of Medicine and Therapeutics, Carol and Richard Yu Peritoneal Dialysis Research Centre, Prince of Wales Hospital, The Chinese University of Hong Kong, Shatin, Hong Kong, China

**Keywords:** Diseases, Nephrology

## Abstract

Malnutrition is a common complication in the dialysis population, both hemodialysis and peritoneal dialysis (PD). We report our exploratory study on the characteristics of intestinal microbiota and nutritional status in PD patients. The nutritional status of our PD patients were evaluated, and their feces were collected for *16S* rRNA gene V3-V4 regions amplification and high-throughput sequencing. The characteristics and differences of microbiota between the well-nourished (W) and malnourished (M) groups were compared. We studied the genera and the operational taxonomic units (OTUs) within the genus of our patients, initially comparing the malnourished and the well- nourished groups and later on reanalyzing the whole group using these OTUs. At the OTU level, 6 bacteria were significantly correlated with the serum albumin level. The abundances of 2 OTUs (OTU208 *Lachnospiraceae_incertae_sedi* and OTU4 *Bacteroides*) were more in W group. Meanwhile, 4 OTUs (OTU225 *Akkermansia*, OTU87 *Megasphaera*, OTU31 *Peptostreptococcaceae_incertae_sedi* and OTU168 *Clostridium_sensu_strictu*) displayed higher abundance among individuals in M group. Notably, the OTU168 *Clostridium_sensu_stricto* was the only bacteria that significantly correlated with serum albumin (r = − 0.356, *P* = 0.05), pre-albumin (r = − 0.399, *P* = 0.02), and SGA (r = 0.458, *P* = 0.01). The higher the OTU168 *Clostridium_sensu_strictu,* the lower serum albumin and pre-albumin and a higher score of SGA signifying a worse nutritional status. Our preliminary findings suggested a relationship between the nutrition status and microbiota in PD patients. Our results provide a basis for further exploration of the interactions between malnutrition and intestinal flora in PD patients with potential interventions using probiotics and prebiotics.

## Introduction

Peritoneal dialysis (PD) is a standard modality of kidney replacement therapy for patients with end-stage kidney disease (ESKD) with increasing use worldwide^1^. Approximately 11% of patients with ESKD are treated with PD in China^2^. Malnutrition is a common complication in patients undergoing PD and the incidence ranges from 18 to 75%^3,4^. The malnutrition is a consequence of a complex mechanism related to multiple factors including not only patients factors such as loss of residual kidney function, intestinal dysfunction^5^ and inadequate nutritional intake^6^, but also factors associated with PD per se such as inadequacy of dialysis, protein loss with PD fluid and continuous micro-inflammation status^7^. Malnutrition is also a key factor that affects patient quality of life and prognosis^3,8,9^. Patients receiving PD who become malnourished have higher hospitalization rates and longer hospital stays, as well as significantly elevated risks of peritonitis and death^10,11^. Therefore, malnutrition in these patients deserves more attention in clinical practice.

The disturbance of gut microbiota in patients with chronic kidney disease (CKD), especially ESKD, has gained increasing attention in recent years. Dysbiosis in CKD patients generally manifests as a loss of beneficial microorganisms and an increase in pathogenic microorganisms and conditional pathogens^12,13^. Disturbance of the gut microbiota may upset gut homeostasis and result in abnormal nutrient absorption and even malnutrition in patients undergoing PD^14^.

The objective of this study is to investigate the composition and distribution characteristics of gut microbiota in patients undergoing PD. We particularly looked into the operational taxonomic units (OTUs) of the microbiome and their relationships with nutritional status. We hope this can provide a scientific basis for the exploration of potential intervention to alleviate malnutrition in this patient cohort in future.

## Materials and methods

### Participants

#### Patient cohort

Patients who underwent continuous ambulatory peritoneal dialysis (CAPD) at the Peritoneal Dialysis Center, Department of Nephrology, General Hospital of Ningxia Medical University between May 2017 and Dec 2018 were included. The inclusion criteria were as follows: (1) age ≥ 18 years; (2) stable CAPD treatment for ≥ 3 months^15^; (3) able to independently collect fecal samples; and (4) voluntary participation in the study with provision of written informed consent. Patients were excluded for the following reasons: (1) cognitive or hearing impairment; (2) other concomitant severe diseases, such as active tuberculosis, malignant neoplasms, severe hepatitis, liver cirrhosis, and active autoimmune disease or other chronic diseases; (3) administration of oral or intravenous antibiotics, microecological preparations, or lactulose within two weeks before sample collection; (4) transfer to other centers on a temporary or long-term basis during the course of PD treatment; (5) poor compliance and the inability to adhere to dialysis prescriptions and execute sample collection instructions; (6) major gastrointestinal surgery within the last 3 months; (7) gastrointestinal endoscopy within the last month; (8) gastrointestinal bleeding within the last month; (9) severe cardiac dysfunction (New York Heart Association class IV); and (10) confirmed diagnosis of PD-related peritonitis, lung infection, or acute infection of other organs or tissues within the last month.

The cut off serum albumin level of 35 g/L as suggested by Chinese National Renal Data System (CNRDS)^16^ was used to divide the CAPD group to the well-nourished group (W group) and the malnourished group (M group).

### Study methods

#### Study design

A cross-sectional observational study design was adopted. The study was approved by the medical research ethics committee of Ningxia Medical University (Approval No.: 2016-254). We confirmed that all methods were performed in accordance with the relevant guidelines and regulations.

#### Clinical data

The following patient information was collected: age, sex, PD vintage, body mass index (BMI), primary disease (chronic glomerulonephritis, diabetes mellitus, or hypertension), biochemical parameters and 24-h residual urine volume. Subjective Global Assessment (SGA) was used to classify for every CAPD patients to level A, B, and C successively^17^. BMI was calculated using the following formula: BMI = Body weight (kg)/height^2^ (m^2^).

Multi-frequency body composition measurement (BCM, Fresenius, German) were performed to measure the nutrition status and hydration status for each participant^18^. The lean tissue index (LTI) and fat tissue index (FTI) normalized to 1.73m^2^ body surface area (kg/m^2^) were used to present muscle and fat content of patient respectively. The item of overhydration (OH) were used to determine fluid status, with a normal reference between plus and minus 1.

#### Dialysis regimen

Patients who underwent PD at our center received PD treatment using the PD-2 system (Baxter, USA) with a daily peritoneal dialysate dose of 6L to 10L. Dialysis was performed with a lactate-buffered dialysate with 2.5 or 1.5% glucose concentration, 132 mmol/L sodium, 1.75 or 1.25 mmol/L calcium, 0.25 mmol/L magnesium, 96 mmol/L chlorine, and 40 mmol/L lactate.

#### Sequencing of gut microbiota

Each fecal sample collected was placed in an ice box immediately and transported to a − 80 °C freezer within 30 min to store until use. Genomic DNA of each fecal sample were extracted with the E.Z.N.A. Stool DNA Kit (Omega Bio-tek, Inc., GA). The V3–V4 regions of 16S rRNA genes were amplified using primer 341F and 805R (341F: 5’-CCTACGGGNGGCWGCAG-3’; 805R: 5’- GACTACHVGGGTATCTAATCC-3). After purification, the polymerase chain reaction products from each sample were indexed and mixed at equal ratios for sequencing by Shanghai Mobio Biomedical Technology Co. Ltd. through the MiSeq platform (Illumina Inc., USA). The total sequencing readouts was 941,946 and mean readouts was 31,398 for each sample.

### Bioinformatics and statistical analysis

Numerical data are expressed as mean ± standard deviation, and categorical data are expressed as number and percentage. Intergroup comparisons of numerical and categorical data were performed using the independent samples t-test and Fisher’s exact test. Statistical analysis was performed using SPSS 20.0 (IBM, USA), with differences considered statistically significant when *P* was < 0.05.

To our knowledge, there is no report on relationship between nutrition status and microbiota in PD patients. Since the present study was exploratory, no reference available for the primary study end-point in calculation of sample size.

For analysis of sequence data, clean data were extracted from the raw data using USEARCH 8.0 (http://drive5.com/usearch) based on the following criteria: (1) the sequences of each sample were extracted by allowing zero mismatches in each index; (2) sequences with overlap < 16 bp were discarded; (3) sequences with an overlap error rate > 0.1 were discarded; and (4) sequences with a length < 400 bp after merging were discarded. The optimized sequences obtained after processing were clustered into operational taxonomic units (OTUs) using UPARSE (version 7.1, http://drive5.com/uparse/), as follows: (1) non-repetitive sequences were extracted from the optimized sequences; (2) single-copy non-repetitive sequences were deleted; (3) OTU clustering was performed using a similarity threshold of 97%; and (4) the representative OTU sequences were generated. Using a confidence threshold of 70%, the representative OTU sequences were compared with sequences in the SILVA database (SSU123, http://www.arb-silva.de) to determine the taxonomic position of each *16S* rRNA sequence. After the OTUs were binned with the UPARSE pipeline, we counted the gross OTUs at each taxonomic level (phylum, class, order, family, and genus). The compositions of gut microbiota at each taxonomic level in two groups were compared by using the Mann–Whitney *U* test.

After obtaining the microbiota that significantly differs between the well-nourished and the malnourished patients, we used Spearman correlation analysis to investigate the whole group of 30 CAPD patients with the effect of those bacteria through both genus and OTU level on the nutritional status of the patients including the serum albumin and prealbumin and SGA. We also look into the correlation of those bacteria with residual kidney function (RKF) and overhydration.

### Institutional review board statement

The study was reviewed and approved by the General Hospital of Ningxia Medical University institutional review board.

### Informed consent

All the participants provided a written informed consent.

## Results

### Analysis of clinical data

#### Patient characteristics

In total, 30 CAPD patients were enrolled with 19 in W group and 11 in M group. The mean patient age in the entire CAPD patient group was 46.3 ± 13.6 years, and there were 12 men (40%). The mean vintage was 26.6 ± 19.8 months. Sixteen patients (53.3%) had primary glomerulonephritis, 6 (20%) had diabetes mellitus, and 8 (26.7%) had hypertensive nephropathy.

Table 1 shows the characteristics of the patients in the M and W group. Among the 30 patients, 19 (63.3%) were in the W subgroup and 11 (36.7%) were in the M subgroup. The M subgroup had more female, a lower residual urine volume, lower hemoglobin level, poorer SGA rating, and more severe overhydration. Differences in age, vintage of PD, primary disease and BMI between the two subgroups were not statistically significant (*P* > 0.05).

**Table 1:** Clinical characteristics of the continuous ambulatory peritoneal dialysis (CAPD) patients.

Parameter	All patients (n = 30)	W group (n = 19)	M group (n = 11)	*P*-value
Male (n, %)	12 (40.0%)	9 (47.4%)	3 (27.3%)	0.021
Age (years)	46.33 ± 13.64	45.05 ± 11.87	48.55 ± 16.65	0.509
Mean vintage (months)	26.63 ± 19.76	24.00 ± 18.47	31.18 ± 21.97	0.346
Primary disease				0.982
Chronic glomerulonephritis	16 (53.3%)	10 (52.6%)	6 (54.5%)	
Diabetes mellitus	6 (20.0%)	4 (21.1%)	2 (18.2%)	
Hypertension	8(26.7%)	5 (26.3%)	3 (27.3%)	
BMI (kg/m^2^)	22.02 ± 2.94	21.72 ± 3.23	22.53 ± 2.43	0.480
Residual urine volume (mL/24 h)	753.00 ± 611.35	933.68 ± 664.498	440.91 ± 346.28	0.013^a^
Dialysis dose (L)	8.2 (6.1, 9.5)	8.5 (6.3, 9.5)	8.8 (6.0, 9.9)	0.86
SGA rating				0.03^a^
A	26 (86.7)	18 (94.7%)	8 (72.7%)	
B + C	4 (13.3)	1 (5.3%)	3 (27.3)	
HGB (g/L)	119.17 ± 15.73	125.11 ± 12.75	108.91 ± 15.55	0.004^a^
ALB (g/L)	37.28 ± 5.46	40.73 ± 2.78	31.32 ± 3.31	0.000
Scr (µmol/L)	825.00 ± 256.52	849.16 ± 234.52	783.26 ± 298.00	0.507
SUA (µmol/L)	385.20 ± 79.00	400.42 ± 76.00	358.91 ± 80.64	0.169
TG (mmol/L)	2.29 ± 1.39	2.59 ± 1.36	1.80 ± 1.36	0.141
LDL (mmol/L)	2.15 ± 0.64	2.08 ± 0.62	2.27 ± 0.67	0.457
OH (L)	3.05 ± 2.59	2.06 ± 1.22	4.66 ± 3.41	0.032^a^
LTI (kg/m^2^)	12.47 ± 2.97	12.63 ± 2.40	12.20 ± 3.84	0.711
FTI (kg/m^2^)	8.23 ± 2.98	8.25 ± 2.83	8.19 ± 3.34	0.960

*W group* Well-nourished group, *M group* Malnourished group**,** *BMI* body mass index, *SGA* subjective global assessment of malnutrition, *HGB* hemoglobin, *ALB* serum albumin, *Scr* serum creatinine, *SUA* serum uric acid, *TG* triglycerides, *LDL* low-density lipoproteins, *OH* overhydration, *LTI* lean tissue index, *FTI* fat tissue index.

^a^*P* < 0.05 indicates statistically significant differences. Intergroup comparisons of numerical and categorical data were performed using the independent samples t-test and Fisher’s exact test. Data are expressed as χ ± s unless otherwise indicated.

#### Gut microbiota in the CAPD patients with different nutritional status

Totally, there were 355 OTUs across the M and W groups, with 35 OTUs unique to the M group, 36 OTUs unique to the W group, and 284 common OTUs to both groups. The beta diversity analysis performed between M and W group showed a significant difference on the general composition in the two groups (*P* = 0.03). (PERMANOVA and PCoA plot in the supplemented files) (Supplementary Figure 1).

**Figure 1:**
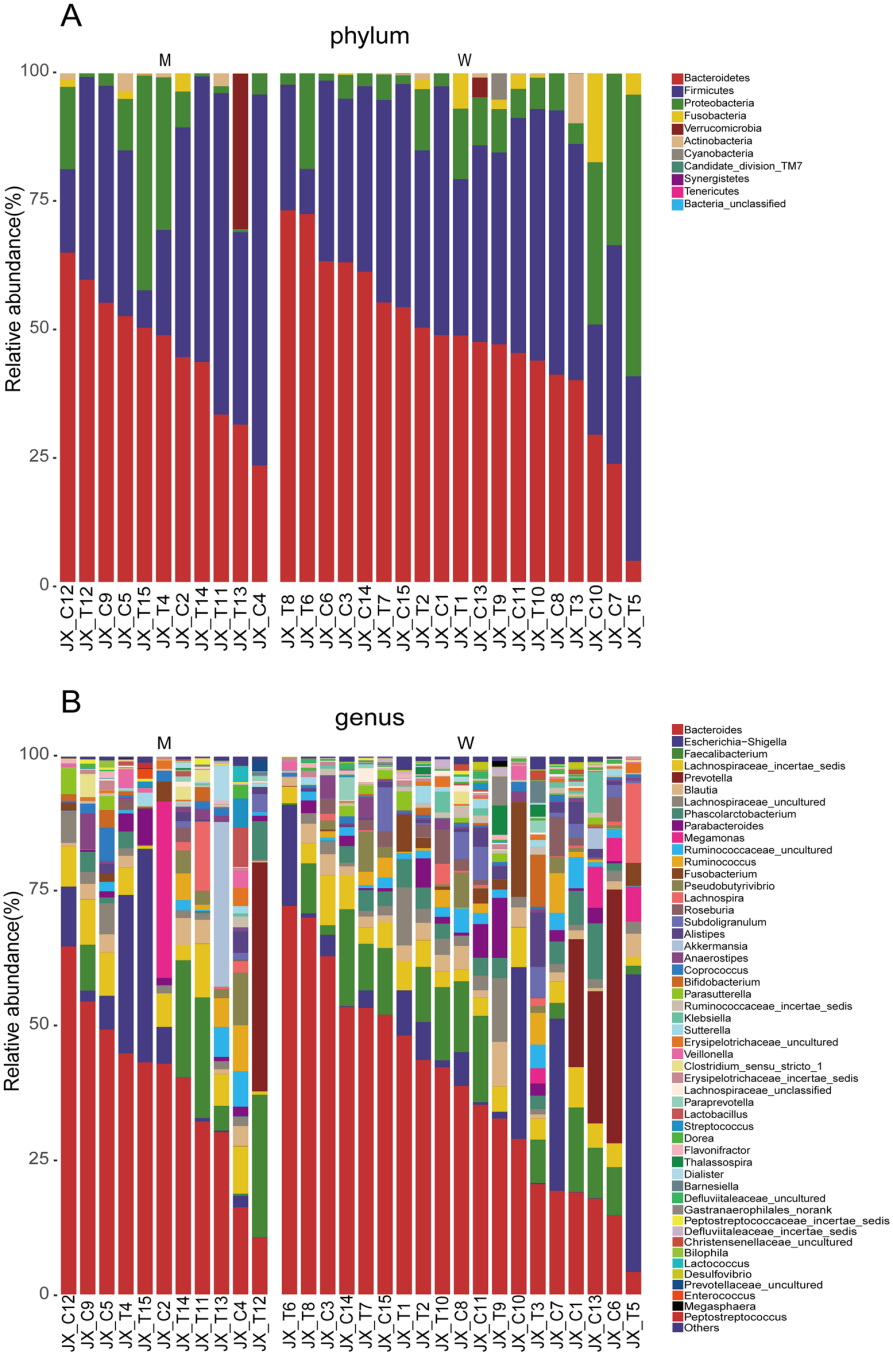
Phylogenetic profiles of the fecal microbiota between the M and W groups of the CAPD patients. (**A**): phylum level; (**B**): genus level. *CAPD* continuance ambulatory peritoneal dialysis, *W* well-nourished group, *M* malnourished group. (Fig. 1 was produced by the R Project for Statistical Computing (r-project.org) using the Grammar of Graphics ggplot2 (tidyverse.org)^44^.

At the phylum level, *Bacteroidetes, Firmicutes,* and *Proteobacteria* were the dominant bacteria of both the M and W groups (Fig. 1A). The abundance of *Bacteroidetes, Firmicutes* and *Proteobacteria* was not different significantly in both groups. At the genus level, the abundances of *Bacteroides, Faecalibacterium*, *Escherichia − Shigella*, and *Lachnospiraceae_incertae_sedis* were the primary genus in both groups, however with no statistical difference between two groups (Fig. 1B).

**Figure 2:**
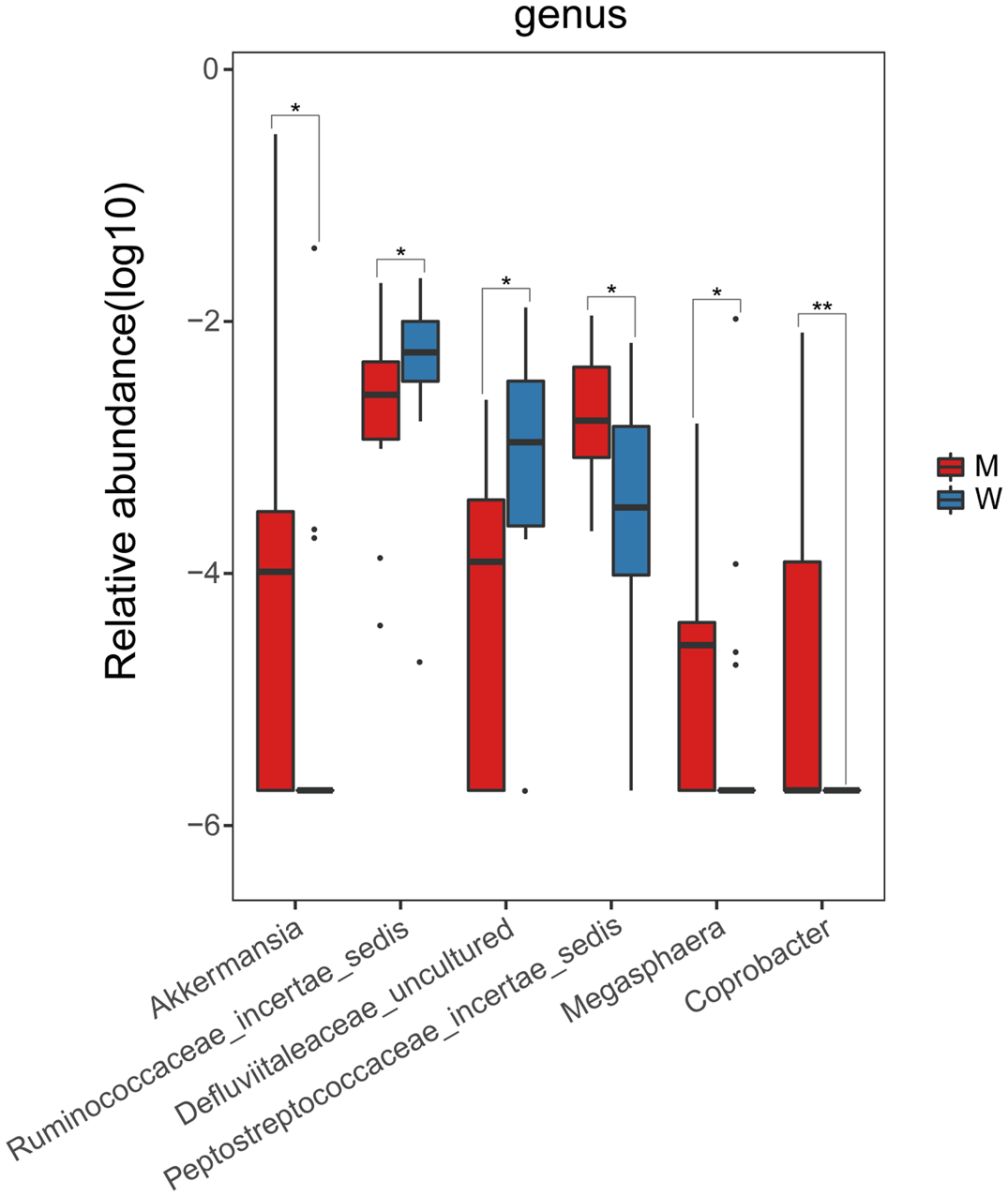
Different abundant bacteria on genus level between the M and W groups of the CAPD patients. *W* well-nourished group, *M* malnourished group.

The abundances of 6 genera, including *Akkermansia, Defluviitaleaceae_uncultured, Megasphaera, Peptostreptococcaceae_incertae_sedis, Ruminococcaceae_incertae_sedis,* and *Coprobacter* were significantly different between the two groups (*P* < 0.05, Wilcoxon rank-sum test) (Fig. 2). The abundances of *Akkermansia, Peptostreptococcaceae_incertae_sedis, Megasphaera* and *Coprobacter* were significantly higher in the M group while *Defluviitaleaceae_uncultured* and *Ruminococcaceae_incertae_sedis* were significantly lower in the M group (Fig. 2).

**Figure 3:**
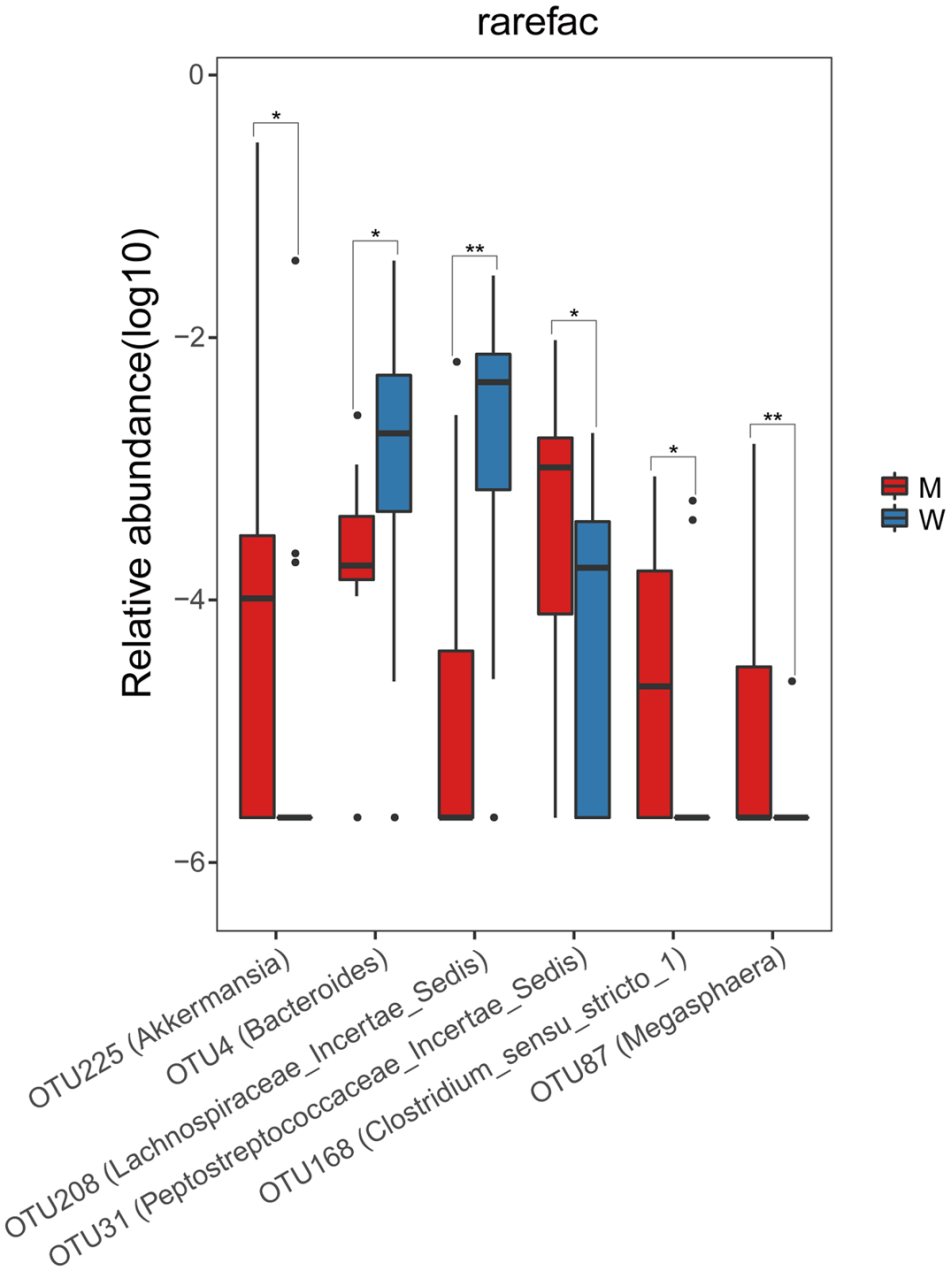
Different abundant bacteria on OTU level between the M and W groups of the CAPD patients. *W* well-nourished group, *M* malnourished group.

Further OTU level comparisons showed that the abundances of 2 OTUs were more in W group, which both displayed higher abundance (*OTU208 Lachnospiraceae_incertae_sedis*: mean log_2_ fold change 2.66, p_adj_ = 0.013 and *OTU4 Bacteroides*: mean log_2_ fold change 3.91, p_adj_ = 0.025, respectively). Meanwhile, 4 OTUs displayed higher abundance among individuals in M group (*OTU225 Akkermansia:* mean log_2_ fold change 3.77, p_adj_ = 0.025; *OTU168 Clostridium_sensu_stricto:* mean log_2_ fold change 1.53, p_adj_ = 0.03; *OTU31 Peptostreptococcaceae_incertae_sedis*: mean log_2_ fold change 2.24, p_adj_ = 0.04, *OTU87 Megasphaera*: mean log_2_ fold change 7.36, p_adj_ = 0.02, respectively). (Table 2 and Fig. 3).

**Table 2:** Differentially abundant bacteria on OTU level between the M and W groups in CAPD patients.

	Mean (M)	SE (M)	Mean (W)	SE (W)	Log_2_ fold change	p-value	p-adjust
OTU225 (Akkermansia)	2.79E − 02	2.77E − 02	2.04E − 03	2.02E − 03	− 3.77	0.014	0.025
OTU31 (Peptostreptococcaceae_incertae_sedis)	1.93E − 03	8.82E − 04	4.09E − 04	1.45E − 04	− 2.24	0.040	0.040
OTU87 (Megasphaera)	2.08E − 04	1.46E − 04	1.26E – 06	1.26E − 06	− 7.36	0.006	0.020
OTU168 (Clostridium_sensu_strictu)	1.49E − 04	8.12E − 05	5.14E − 05	3.59E − 05	− 1.53	0.022	0.026
OTU208 (Lachnospiraceae_incertae_sedis)	8.94E − 04	6.08E − 04	5.68E − 03	1.60E − 03	2.66	0.002	0.013
OTU4 (Bacteroides)	5.09E − 04	2.22E − 04	7.65E − 03	2.86E − 03	3.91	0.017	0.025

*CAPD* continuous ambulatory peritoneal dialysis, *W* well-nourished subgroup, *M* malnourished subgroup.

Log_2_ fold change was calculated by using W/M taking 2 as the logarithm of the base.

#### Correlation of abundant bacteria on OTU levels with nutrition in the whole group of 30 CAPD patients

In order to analyse more the effects of the different OTUs, we study the effects of these 6 OTUs on the nutritional and other clinical parameters in the whole group of 30 CAPD patients. In this whole group of 30 CAPD patients, 6 bacteria at the OTU level were significantly correlated with the serum albumin level, among which 4 presented negative correlation (OTU225 *Akkermansia,* OTU31 *Peptostreptococcaceae_incertae_sedis,* OTU87 *Megasphaera*, OTU168 *clostridium_sensu_stricto*) while 2 presented positive correlation (OTU208 *Lachnospiraceae_incertae_sedis* and OTU4 *Bacteroides*) (Table 3). OTU87 *Megasphaera* and OTU168 *Clostridium_sensu_stricto* were both negatively correlated with the pre-albumin.

**Table 3:** Correlations of abundant bacteria on OTU level with clinical parameters in the whole group of 30 CAPD patients.

Rho	Gender	Age	PD Vintage	Hemoglobin	Serum albumin	Pre-albumin	Residual urine volume	SGA	OH
OTU225 (Akkermansia)	0.126	0.321	0.279	− 0.122	− 0.495^▲^	− 0.298	− 0.342	0.027	0.434*
OTU31 (Peptostreptococcaceae_incertae_sedis)	0.048	0.107	− 0.160	− 0.020	− 0.394*	− 0.293	− 0.177	0.218	0.126
OTU87 (Megasphaera)	0.208	0.181	0.391*	− 0.288	− 0.446*	− 0.479^▲^	− 0.260	0.291	0.438*
OTU168 (Clostridium_sensu_stricto)	0.066	0.360	0.101	− 0.078	− 0.356^★^	− 0.399*	− 0.264	0.458*	0.064
OTU208 (Lachnospiraceae_incertae_sedis)	− 0.344	− 0.061	− 0.278	0.162	0.603^#^	0.207	0.425^▲^	− 0.150	− 0.434*
OTU4 (Bacteroides)	− 0.173	− 0.403*	− 0.534^▲^	0.227	0.392*	0.219	0.433*	− 0.243	− 0.254

*PD* peritoneal dialysis, *SGA* subjective global assessment, *OH* overhydration.

*P < 0.05, ^★^*P* = 0.05, ^▲^*P* < 0.01, ^#^*P* < 0.001.

Notably, the OTU168 *Clostridium_sensu_stricto* was the only bacteria that significantly correlated with serum albumin (r = − 0.356, p = 0.05), pre-albumin (r = − 0.399, p = 0.02), and SGA (r = 0.458, p = 0.01). The higher the OTU168 *Clostridium_sensu_stricto*, the lower serum albumin and pre-albumin and also a higher score of SGA which also signified a worse nutritional status.

**Figure 4:**
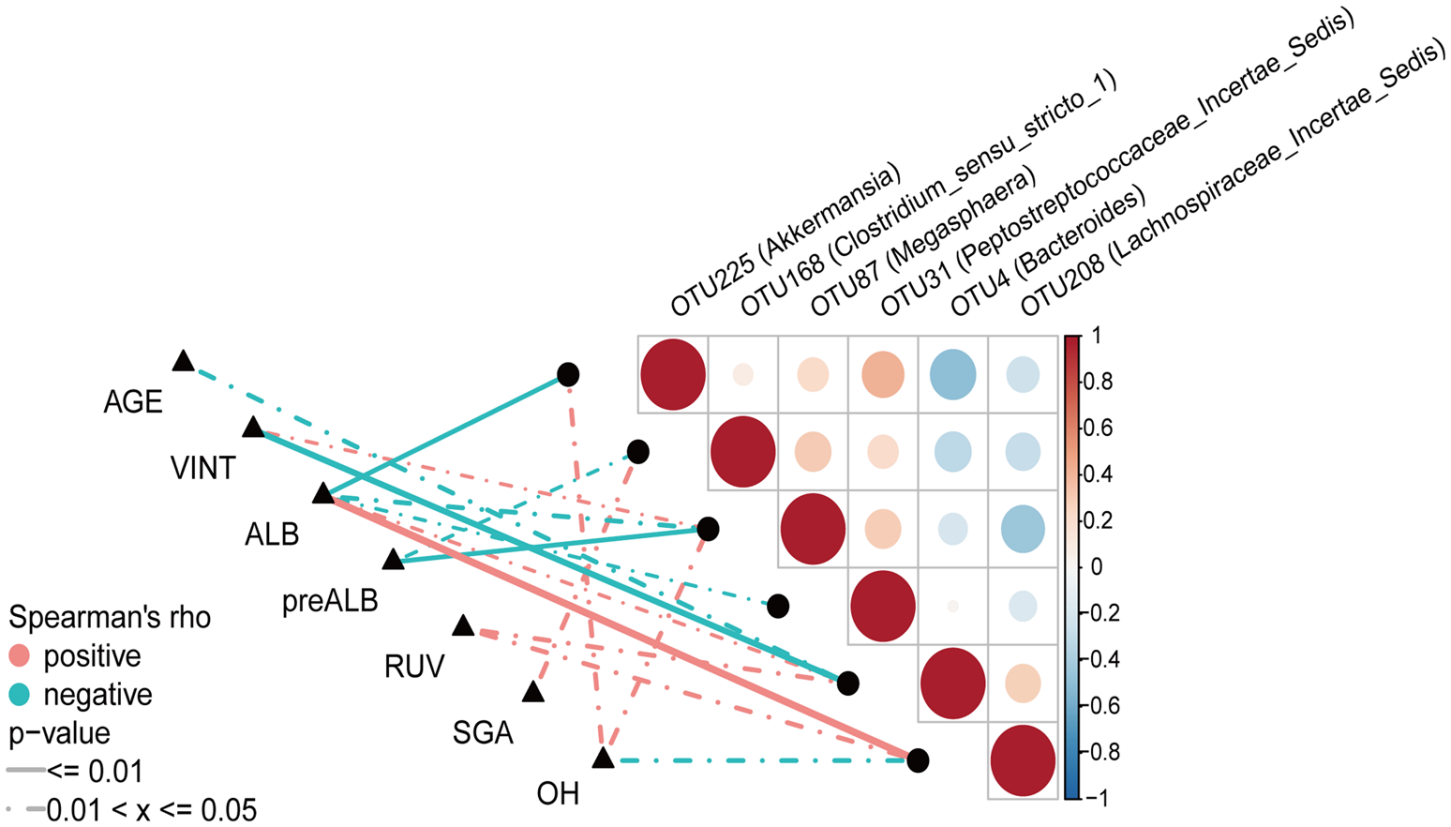
Correlation of abundant bacteria on OTU level with nutrition in the whole group of 30 CAPD patients based on Spearman correlation analysis. *CAPD* continuance ambulatory peritoneal dialysis, *VINT* vintage, *ALB* albumin, *preALB* pre-albumin, *RUV* residual urine volume, *SGA* subjective global assessment, *OH* over hydration.

In Table 1, there are other clinical characteristics which showed statistical difference at baseline between M and W group. When we studied the correlation between gut bacteria at OTU level and those clinical characteristics in the whole group of 30 CAPD patients, it also helped to clarify the possible confounders’ effects. The OTU208 *Lachnospiraceae_incertae_sedis* was related to residual urine volume and overhydration besides albumin level. The OTU4 *Bacteroides* was also correlated with multi-items including age, vintage, and residual urine volume. The OTU225 *Akkermansia* was positively correlated with overhydration and negatively with serum albumin. OTU87 *Megasphaera* was also positively correlated with dialysis vintage and overhydration while having a negative correlation with albumin and pre-albumin. OTU31 *Peptostreptococcaceae_incertae_sedis* had a negative correlation with serum albumin and did not show any significant correlation with other confounding factors. Finally, the OTU168 *Clostridium_sensu_stricto* was significantly associated with nutrition markers presented as albumin, pre-albumin and SGA. Meanwhile, it did not show any significant correlation with other confounding factors. Table 3 and Fig. 4 sum up the relationship of these 6 OTUs with the nutritional and clinical parameters in these 30 CAPD patients.

## Discussion

Recent research on the relationship between intestinal microecology and chronic diseases suggests the presence of severe dysbiosis within the gut microbiota of ESKD patients, which is associated with disease progression and the onset and development of complications^19,20^. In the present study, sequencing analysis was performed on the gut microbiota of patients undergoing PD, and the gut microbiota characteristics of patients with different nutritional statuses were compared. Our results indicated that there were differences in the species abundance and gut microbiota compositions of patients undergoing CAPD based on nutritional status, suggesting that changes in bacterial taxa and microbiota structure might be related to malnutrition.

Physical, chemical, biological, and immune barriers exist in normal intestinal mucosa. In patients undergoing PD, several factors may lead to ischemia, edema and injury in the mucosa which can damage the intestinal barrier and result in gastrointestinal dysfunction and gut microbiota dysbiosis^21^. The increased intraperitoneal pressure, long-term retention of dialysate in the peritoneal cavity and decreased gastrointestinal motility caused by PD together with the use of iron supplements and antibiotics can damage the intestinal barrier, leading to changes in the abundance and diversity of gut microbiota^22–24^.

Previous studies reported changes in the gut microbiota composition among patients with CKD and ESKD^25,26^. Our study is probably the first focusing on the relationship between gut microbiota and nutritional status in patients undergoing PD. Studies indicated a gut microbiota imbalance in patients undergoing PD and this imbalance is characterized by a decrease in beneficial bacterial populations and an increase in pathogenic or conditional pathogenic bacteria^27–29^. In the present study, OTUs across the M and W CAPD subgroups showed that the fecal microbiota of the two subgroups had similar numbers of bacteria but different bacterial populations.

We found that 6 genera, including *Akkermansia, Defluviitaleaceae_uncultured, Megasphaera, Peptostreptococcaceae_incertae_sedis, Ruminococcaceae_incertae_sedis,* and *Coprobacter* were significantly different between the two groups. In a recent study trying to define dysbiosis for a cluster of chronic diseases including urinary stone disease, obesity, diabetes, cardiovascular disease, and kidney disease, each disease state was associated with a loss of microbial diversity in the gut^30^. They reported that among the taxa that were most consistently found to be depleted in disease individuals were the *Bacteroides, Coprococcus, Prevotella, Ruminococcus, and Sutterella*. However, their subsequent analysis suggests that the association of some of these taxa with dysbiosis is closely tied to their number of constituent OTUs in the gut^30^. Some taxa, primarily *Bacteroides, Corynebacterium*, and *Anaerococcus* were identified as dysbiotic more than expected given their diversity, indicative of a more specific physiological interaction between these taxa and disease. It was previously reported that *Bacteroides* either has a health-protective^31^ or health-antagonistic response^32^. Results from their analysis show that this genus is strongly associated with both health and disease in terms of the number of independent populations it was found to be associated with. Thus it is likely that some OTUs within the genus provide more of a protective effect and others more of a detrimental health impact^30^. Thus our analysis focused more on the OTU level of our patients, initially comparing the malnourished and the well- nourished groups and later on reanalyzing the whole group of the 30 CAPD patients. Notebly, the OTU87 *Megasphaera* and OTU168 *Clostridium_sensu_stricto* were both negatively correlated with the serum albumin and pre-albumin. Especially, the OTU168 *Clostridium_sensu_stricto* was the only bacteria that significantly correlated with SGA, pre-albumin and serum albumin at the same time. The higher the OTU168 *Clostridium_sensu_stricto,* the lower serum albumin and pre-albumin and also a higher score of SGA which also signified a worse nutritional status. These two OTUs may have significantly adverse association with the nutritional status of PD patients.

The *Clostridium *sensu stricto are grouped around the type species *Clostridium butyricum* and belong to the *Clostridium* cluster I within the *Clostridiaceae* family. It has been reported that up to 72 *Clostridium* spp. have been detected in the human gastrointestinal samples, of which 30 belong to the *Clostridium *sensu stricto^33^. A recent study suggested that the change in the abundance of *Clostridium *sensu stricto was positively associated with IL-10 and LDL-cholesterol, while a negative correlation was observed with changes in insulin levels^34^. Studies also showed that CD4 + T regulatory cells (Tregs) are abundant in intestinal lamina propria, and their accumulation in the small intestine and colon is differentially regulated. The induction of colonic Tregs is dependent on commensal microorganisms with specialized properties. Among the indigenous commensal bacteria, *Clostridium spp* belonging to clusters IV and XIVa are outstanding inducers of Tregs in the colon^35^. Notably, *Clostridium* clusters IV and XIVa constitute a smaller proportion of the fecal community in patients with human inflammatory bowel diseases than in healthy controls^36^. Another recent study of microbiota analysis on the liver steatosis and fibrosis in obese patients found the relative abundance of fecal *Clostridium *sensu stricto was significantly decreased with the presence of liver fibrosis and was negatively associated with liver stiffness measurement and myosteatosis^37^.

Our finding of OTU168 *Clostridium_sensu_stricto* association with a worse nutrition status in PD patients with a lower serum albumin and pre-albumin and also a higher score of SGA would warrant further investigation. It is of note that this OTU did not show any correlation with residual kidney function or hydration status, pointing that this OTU168 may have a particular significant value in the nutritional status in PD patients.

OTU87 *Megasphaera* was also negatively correlated with the serum albumin and pre-albumin but not with SGA. It also did not show significant correlation with residual kidney function but had a positive correlation with overhydration. The negative association with the albumin and prealbumin may be partly related to the overhydration status in patients with a higher abundance of this OTU87 *Megasphaera*. Despite this, it is of note that human gut isolates of *Megasphaera* have been found to harbor a wide range of carbohydrate active enzymes alongside with oligopeptide transport systems allowing them to use carbohydrates as well as a wide range of amino acids as carbon source^38^. Besides the production of propionate, human gut isolates of *Megasphaera*, have been shown to produce butyrate from glucose^38^.

The importance of OTUs has been studied in a group of pre-diabetes patients. They found that the two OTUs that differed the most were a member of the order *Clostridiales* and *Akkermansia muciniphila*, which both displayed lower abundance among 134 Danish adults with prediabetes when compared with 134 age- and sex-matched individuals with normal glucose regulation^39^. Like what we have performed for the whole group of 30 CAPD patients, they went on further to study the association between differentially abundant OTUs and clinical biomarkers relevant for diabetes in the entire group of 268 individuals^39^. They found that two OTUs (OTU 3856408 and OTU 193129) classified as *Lachnospiraceae*, two OTUs (OTU 4364405 and OTU 819353) classified as *Ruminococcaceae* and OTU 4465124 classified as *Clostridium* correlated particularly strongly and negatively with fasting plasma levels of glucose, insulin, C-peptide, triacylglycerol and hsCRP, as well as HOMA-IR, BMI and waist circumference^39^.

We also found two OTUs (OTU208 *Lachnospiraceae_incertae_sedi* and OTU4 *Bacteroides*) presented positive correlation with serum albumin. OTU4 *Bacteroides* also has a positive correlation with residual kidney function making it difficult to discern the effects whether it is more related to the residual kidney function or the nutritional status per se. It is obvious that PD patients with good residual kidney function can have a good nutritional status^21,40^. Despite such complex interactions, recent review suggested that metabolites secreted by different *Bacteroides spp*. assist in maintaining stability of the immune system^41^. These species are primary producers of short-chain fatty acids in the human gut, mostly in the form of acetate and propionate which are important for the maintenance of intestinal homeostasis^41^. Both acetate and propionate are potent anti-inflammatory mediators as they inhibit the release of pro-inflammatory cytokines from neutrophils and macrophages. Also, butyrate increases expression of tight-junction proteins in the gut to reduce potential gut hyperpermeability. This, in turn, decreases inflammation and endotoxemia that are associated with leaky gut^21,41,42^. It would be interesting to study further the OTU4 *Bacteroides* effect on the immune regulation and endotoxemia in future.

OTU208 *Lachnospiraceae_incertae_sedis* also has a positive correlation with residual kidney function and a negative correlation with overhydration. This adds to the complexity that whether this OTU has a significant relationship with nutritional status especially when overhydration is also having a negative correlation with residual kidney function^40,43^. It would be interesting to study this OTU further in future in a bigger group of PD patients.

In our study, another 2 OTUs showed negative correlation with serum albumin but not with serum pre-albumin or SGA (OTU225 *Akkermansia,* OTU31 *Peptostreptococcaceae_incertae_sedis*). OTU225 *Akkermansia* was shown to have a positive correlation with overhydration. OTU31 *Peptostreptococcaceae_incertae_sedis* showed no relationship with residual kidney function or overhydration. The significance of these 2 OTUs on nutrition required further study.

It should be noted that our findings only provide a snapshot of the complex fecal microbiomes in PD patients and nutritional subgroups, and only the bacterial composition was analyzed. There are several limitations of this study. First, the population in this study comprised patients who were undergoing PD. Dialysis in the peritoneum around the gut may have effects on intestinal functions and environment, thereby affecting microbiota. Additionally, the results suggest that the composition and structure of intestinal microflora are related to nutritional status, but it is not clear whether the relationship is causal. Finally, the small sample size of malnourished patients may have magnified the effects of individual differences.

## Conclusion

In conclusion, this is an exploratory study looking into the association of gut microbiome with nutritional status of patients on peritoneal dialysis. We have focused our study on the OTU level of the microbiota which should have advantage over studies on genus level alone. Significant differences were also found in the compositions of gut microbiota between malnourished and well-nourished patients undergoing PD. In particular, OTU168 *Clostridium_sensu_stricto* was associated with a worse nutritional status in PD patients with a lower serum albumin and pre-albumin and also a higher score of SGA. The results of this study indicate the presence of a possible gut microbiota imbalance in patients undergoing PD with malnutrition. Further research is required to elucidate the association of the gut microbiota with malnutrition in patients undergoing PD and look into the pathogenetic mechanisms by which gut microbiota causing malnutrition in PD patients. This may help in future to study the potential use and effectiveness of probiotic formulations in improving nutritional status in PD patients.

## Supplementary Information

Supplementary Figure 1.

Supplementary Information 1.

## Data Availability

The raw sequence data of the *16S* rRNA gene V3–V4 regions and relevant information are available in NCBI Sequence Read Archive database under accession number PRJNA704190. Other datasets used and/or analyzed during the current study are available from the corresponding author on reasonable request.
