# Supplementary figures and images for: Relationship between gut microbiota and nutritional status in patients on peritoneal dialysis
Source: Sci Rep. 2023 Jan 28;13:1572. doi: 10.1038/s41598-023-27919-3 (PMC9884196; doi:10.1038/s41598-023-27919-3)

PCoA

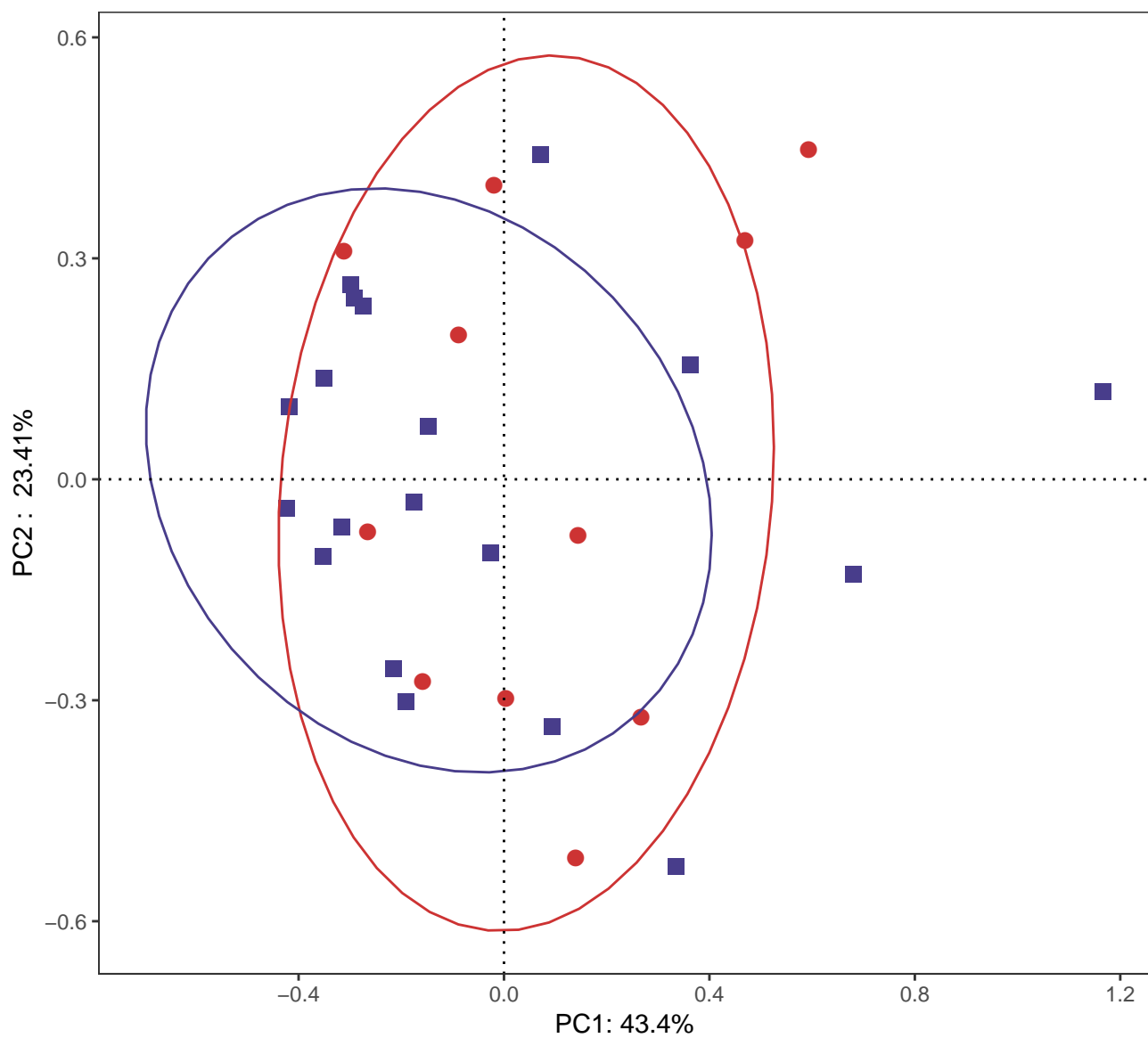

Supplement: Supplementary file 1 — Supplementary Figure 1. [file 41598_2023_27919_MOESM1_ESM.pdf]
